# New Strategies to Combat Human Fungal Infections

**DOI:** 10.3390/jof10120880

**Published:** 2024-12-18

**Authors:** André Luis Souza dos Santos, Marta Helena Branquinha

**Affiliations:** 1 Laboratório de Estudos Avançados de Microrganismos Emergentes e Resistentes (LEAMER), Departamento de Microbiologia Geral, Instituto de Microbiologia Paulo de Góes (IMPG), Centro de Ciências da Saúde (CCS), Universidade Federal do Rio de Janeiro (UFRJ), Rio de Janeiro 21941-902, RJ, Brazil; 2 Rede Micologia RJ—Fundação de Amparo à Pesquisa do Estado do Rio de Janeiro (FAPERJ), Rio de Janeiro 21941-902, RJ, Brazil,

Over the past few decades, numerous reports have highlighted the significant rise in fungal infections worldwide, contributing to considerable morbidity, mortality, and escalating healthcare costs [1,2,3,4,5,6,7,8]. As a result, these infections are increasingly recognized as a major global health threat. The release of the World Health Organization’s (WHO) first ever Fungal Priority Pathogens List in 2022 garnered significant international attention [9]. Although the WHO list remains incomplete, particularly due to the omission of several potential fungal pathogens—especially those endemics to developing countries—this initiative has undeniably underscored the urgent need for emerging public health policies to address this neglected group of microorganisms. In this vein, in early 2024, a publication emphasized the growing importance of fungal infections, presenting alarming estimates regarding the incidence and mortality of the most concerning, invasive forms [8]. Data revealed an estimated annual incidence of 6.5 million cases of invasive fungal infections, which represent the most severe and challenging mycoses to treat, leading to approximately 3.8 million deaths each year [8].

Compounding this issue, the available arsenal for combating fungal infections is critically limited, consisting of only a few antifungal drugs belonging to four main classes: azoles (e.g., fluconazole, itraconazole, voriconazole, posaconazole), polyenes (e.g., amphotericin B, nystatin), echinocandins (e.g., caspofungin, micafungin, anidulafungin), and antimetabolites (e.g., 5-flucytocine). Antifungal drugs are often associated with significant toxicity and a range of harmful side effects [10]. The seriousness of this situation can be better attributed to fungal infections, as their eukaryotic cell architecture and conserved gene sequences are closely related to those of metazoans, including humans. As a result, there are genetic, cellular, biochemical, and immunological similarities between the fungal pathogens and their host cells, making treatment particularly difficult. Resistance to antifungal drugs complicates infection management, leading to prolonged illness, increased healthcare costs, and higher mortality rates. The emergence of antifungal resistance is accelerating at an alarming rate, and, as of now, no vaccine is available to prevent these infections [11]. This crisis is further fueled by the overuse of, misuse of, and indiscriminate reliance on antimicrobial drugs to treat infections, which has led to resistance across a broad spectrum of pathogens, including fungi, on a global scale. The WHO has recognized antimicrobial resistance as one of the most pressing threats to public health, sparking urgent calls for action from global health authorities and the media to address this escalating issue.

Given the alarming rise in fungal infections, particularly those caused by resistant strains, there is an increasing call for the urgent development of innovative strategies to combat these diseases. In this context, identifying novel targets within fungal cells, along with the synthesis and discovery of new bioactive compounds, highlights the critical need for continued research and development in the field of Mycology. The current Special Issue in the *Journal of Fungi*, titled “New Strategies to Combat Human Fungal Infections”, provides a valuable platform for sharing new knowledge, insights, and perspectives on this critical issue. This Special Issue features a total of eight papers, including six research articles, one review, and one case report. To spark the reader’s interest, a brief summary of each published paper is provided below.

Brilhante and colleagues [12] explored the promising anti-*Histoplasma capsulatum* properties of chitosan, a linear polysaccharide derived from partial chitin deacetylation. Their research demonstrated that chitosan exhibited antifungal activity against both the yeast and filamentous forms of *H. capsulatum*, a well-known dimorphic fungus. Notably, their study revealed a synergistic effect when chitosan was combined with either amphotericin B or itraconazole, enhancing its activity against the yeast form. Additionally, mature *H. capsulatum* biofilms were significantly disrupted by chitosan treatment, leading to substantial reductions in both biomass and cell viability. These findings suggest that chitosan holds potential as a therapeutic platform for combating *H. capsulatum* infections.

The paper published by Fusco and colleagues [13] investigated the potential of *Lactiplantibacillus plantarum* as a modulator of the skin damage caused by *Malassezia furfur*. The authors hypothesized that *Lactobacillus* species, which are part of the endogenous microbiota of healthy skin, play a protective role against pathogenic microorganisms. Their results demonstrated that *L. plantarum* effectively mitigated the damage induced by *M. furfur* in human keratinocytes (HaCaT cell line). Their study showed a significant reduction in phospholipase activity, a key virulence factor of the fungus, as well as enhanced wound repair in vitro. This was attributed to the restoration of monolayer barrier integrity and a reduction in inflammasome activation. The findings suggest that *L. plantarum* could be beneficial in topical formulations for improving skin health.

Vahedi-Shahandashti and colleagues [14] addressed a critical and often overlooked issue: the need to continually revise, optimize, standardize, and enhance antifungal susceptibility testing (AFST) methods, mainly for emerging fungal pathogens. The authors focused on the impact of different growth media, as the composition of culture media directly influences the expression of fungal molecules. To explore this, the authors compared AFST results using various complex media and the standard RPMI medium against both *Candida* and *Aspergillus* species, assessing their susceptibility to azoles and polyenes. Their study revealed significant variability in minimum inhibitory concentrations (MICs) depending on the culture medium used, particularly in *Candida* species, with notable differences between the European Committee on Antimicrobial Susceptibility Testing (EUCAST) and E-test methods. This finding serves as a crucial warning for the international scientific community, emphasizing the need to rethink and refine AFST methodologies. Improved testing will provide more accurate data on fungal susceptibility, ultimately guiding clinicians in effective fungal infection management.

The paper of Andrade and colleagues [15] explored the multiple effects of cyclosporine on key virulence factors expressed by *Cryptococcus neoformans* cells. It is well established that cyclosporine inhibits the calcineurin pathway, which is crucial for the positive regulation of many fungal virulence factors. Adopting an antivirulence approach, the authors treated *C. neoformans* cells with subinhibitory concentrations of cyclosporine, revealing significant alterations in fungal morphology. These changes included an increase in lipid bodies and chitin, a key cell wall polysaccharide, as well as a decrease in urease secretion. Additionally, the size and physicochemical properties of the polysaccharide capsule, the primary virulence factor of this fungus, were notably affected.

Sousa and colleagues [16] investigated the potential of coordination compounds containing 1,10-phenanthroline as a ligand and transition metals as potent antifungal agents against *Fonsecaea pedrosoi*, the main etiological agent of chromoblastomycosis. The authors demonstrated the antifungal activity of silver(I)-1,10-phenanthroline complexes and their ability to disrupt key fungal virulence factors. More specifically, the complexes interfered with the (i) filamentation process, preventing the transformation of conidia into hyphae; with (ii) biofilm formation, promoting its disarticulation; and with the (iii) melanin production and (iv) enzymatic activity of both metallo- and aspartyl-type proteases. Notably, the complexes also induced intracellular reactive oxygen species production, thus contributing to their antifungal effectiveness against this opportunistic pathogen.

The article published by Xisto and colleagues [17] explored the potential of international repositories of bioactive molecules in discovering new compounds with antifungal activity against the etiologic agents of mucormycosis. This fungal infection is particularly concerning due to its high mortality rates, difficult diagnosis, and limited treatment options. Their study emphasizes the critical need to expand the search for novel therapeutic agents to effectively combat these invasive infections. In this context, the authors tested the Pandemic Response Box^®^ library (Medicines for Malaria Venture, Geneva, Switzerland), which contains 400 different compounds, against *Rhizopus* species. The results revealed that alexidine and three non-commercial molecules exhibited significant anti-*Rhizopus* activity. These compounds exerted their antifungal effects by inducing oxidative stress, depolarizing the mitochondrial membrane, and causing alterations to essential structures, including the fungal cell wall and plasma membrane. Notably, the selected compounds also demonstrated anti-biofilm activity, further supporting their potential as therapeutic agents for mucormycosis.

Akinosoglou and colleagues [18] authored a comprehensive review of amphotericin B, a polyene antifungal agent that has been extensively used to treat severe systemic fungal infections. Despite its proven efficacy, amphotericin B is associated with significant side effects, including nephrotoxicity, electrolyte imbalances, and infusion-related reactions. To address these challenges, lipid formulations of amphotericin B have been developed to reduce toxicity while maintaining its therapeutic effectiveness. This review paper thoroughly examines these aspects, as well as potential future adaptations of amphotericin B, ensuring its continued use in clinical practice.

Leng and colleagues [19] presented a clinical case highlighting the successful use of a combined antifungal therapy involving oral isavuconazole, nebulized amphotericin B, and bronchoscopic administration of amphotericin B to treat a 70-year-old male patient diagnosed with pulmonary mucormycosis caused by *Rhizopus microsporus*. In addition to the case report, the authors conducted a review of retrospective cases from the literature to explore the effectiveness of these treatment strategies for managing this challenging infection.

In summary, this Special Issue presents a collection of high-quality papers in the field of Medical Mycology, with a particular focus on alternative treatments for fungal infections. The editors sincerely hope that these papers, as well as the Special Issue in its entirety, will inspire enthusiasm and stimulate scientific curiosity among young students and researchers worldwide. The editors would like to express their heartfelt gratitude to all the contributing authors for their invaluable collaboration, which has enriched the field with new insights and helped bring Mycology studies into the spotlight.

## Data Availability

Not applicable.
